# Transient pain and long-term gain: adjuvant dose directs immune memory

**DOI:** 10.1172/JCI190524

**Published:** 2025-04-15

**Authors:** Pabitra B. Pal, Smita S. Iyer

**Affiliations:** 1 Department of Pathology, School of Medicine, University of Pittsburgh, Pittsburgh, Pennsylvania, USA.; 2 California National Primate Research Center, UCD, Davis, California, USA.

## Abstract

Vaccine hesitancy is often fueled by fears of side effects; however, most reactions result from innate immune activation and cytokine production, which are required for lasting immunity. For effective vaccines against HIV, innate activation is essential for differentiation of CD4^+^ T cells into T follicular helper cells (T_FH_), which guide rare B cells to mature into long-lived plasma cells that produce durable neutralizing antibodies (nAbs). In this issue of the *JCI*, Parham Ramezani-Rad et al. show that higher doses of saponin QS-21–MPLA nanoparticle (SMNP) adjuvant, combined with BG505 MD39 envelope (Env) protein, enhanced cytokine responses, drove stronger Env-specific T_FH_ responses in blood, and increased Env-specific bone marrow plasma cells compared with lower doses. While tier 2 nAbs were sustained at memory in only a subset of animals, predominantly at the highest adjuvant dose, these findings highlight transient reactogenicity as an essential mechanism — not a flaw — for building durable immune memory.

## Adjuvants help the immune system help itself

The immune system distinguishes self from non-self with remarkable precision, even down to single amino acids; but peptide and protein antigens alone do not elicit robust immune responses on their own, because they lack danger signals — pathogen-associated molecular patterns (PAMPs) — needed to activate pattern recognition receptors (PRRs) on innate immune cells (1, 2). These receptors initiate the signals required for T and B cell activation (3, 4). Adjuvants drive innate immune activation by engaging PRRs on antigen-presenting cells (APCs), such as dendritic cells, thereby enabling effective T and B cell responses (5–7). Subsequent CD4^+^ Th1 cell responses support memory CD8^+^ T cell development, while CD4^+^ T follicular helper (T_FH_) cells help B cells proliferate and mature in germinal centers (GCs) into memory B cells and long-lived, antibody-secreting plasma cells (8).

**Figure 1:**
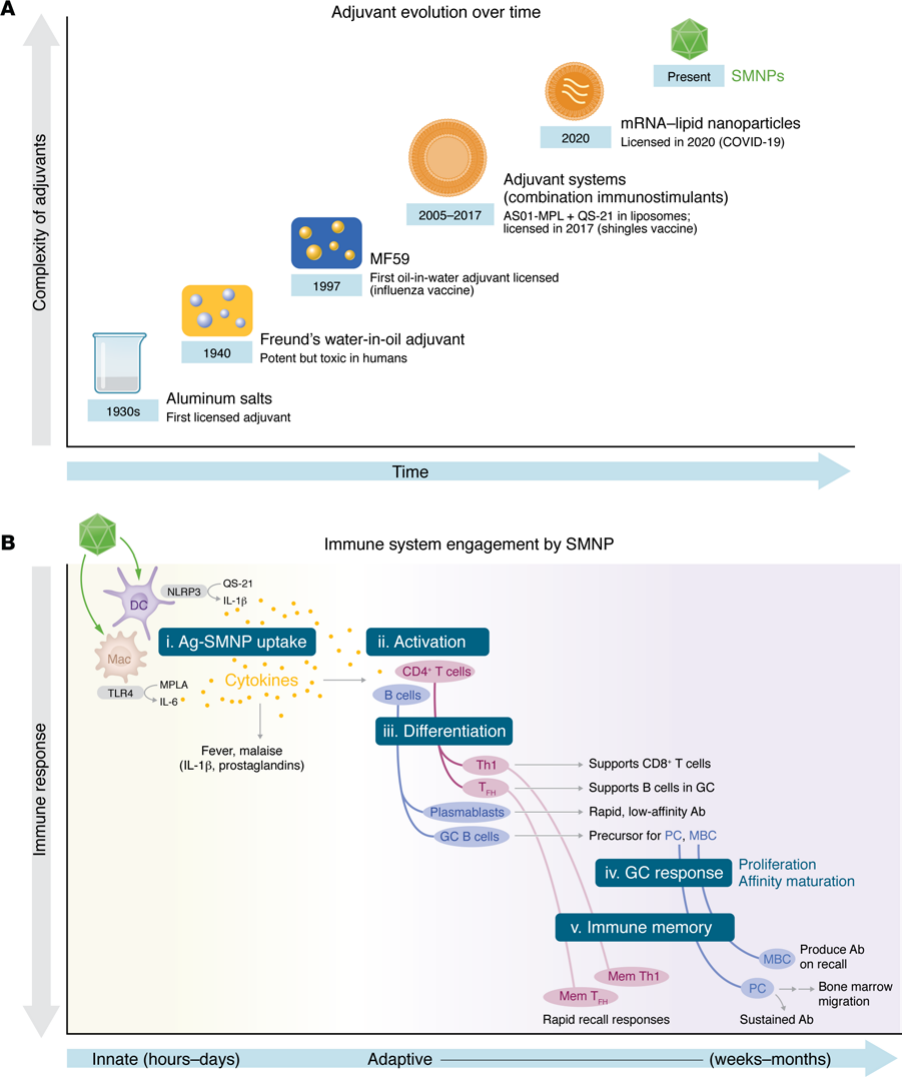
Vaccine adjuvants have evolved since 1930 and now include saponin and MPLA nanoparticles (SMNPs). (**A**) The evolution of vaccine adjuvants started with aluminum salts as the first licensed adjuvant and has advanced to include systems such as SMNP — which integrates the TLR4 agonist MPLA and the saponin QS-21 and has cage-like nanoparticle structure — to amplify immune responses. (**B**) (i) SMNP engages the immune system by activating APCs via TLR4 and NLRP3 inflammasome pathways, leading to the release of inflammatory cytokines (e.g., IL-1β, IL-6), which drive innate immune responses and induce systemic effects such as fever. Mac, macrophage; DC, dendritic cell. (ii) These signals activate CD4^+^ T cells and B cells. (iii) Differentiation into Th1 cells supports CD8^+^ T cell responses for intracellular pathogen clearance and T_FH_ that provide critical support for B cells in GCs. B cells differentiate into plasmablasts and GC B cells. (iv) B cells proliferate and undergo affinity maturation. (v) Long-lived memory B cells (MBC) and long-lived plasma cells (PC) that secrete antibodies contribute to durable immunity. Memory T cells support rapid recall responses.

First introduced in the 1930s, aluminum salts (Alum) — including aluminum potassium sulfate, aluminum hydroxide, and aluminum phosphate — have enhanced antibody responses to diphtheria toxoid and remain cornerstone components of several licensed vaccines (9). In the 1940s, complete Freund’s adjuvant (CFA), an oil-in-water emulsion containing mycobacterial components, was developed. Although CFA is highly potent, toxicity limited its use to experimental autoimmunity models (10, 11). In 1997, the oil-in-water emulsion MF59 was licensed for influenza vaccines for older adults, offering a safer alternative (12, 13). The demand for effective adjuvants capable of eliciting immunity against challenging pathogens such as HIV drove the development of advanced systems, such as AS01, a lipid-based carrier designed to integrate multiple immunostimulatory molecules. AS01, used in the herpes zoster (shingles) vaccine for adults aged 50 and older (14), combines two components: monophosphoryl lipid A (MPLA), a TLR4 agonist that augments APC responses, including in B cells; and QS-21, a saponin that enhances antigen presentation by activating NLRP3 inflammasomes (15) (Figure 1).

Adjuvants such as army liposomal formulation Q (ALFQ), similar in composition to AS01, have shown stronger CD4^+^ T_FH_ cell responses and memory antibody generation in macaques compared with MPLA-alum formulation (ALFA) (15, 16). Likewise, CAF01, a liposomal adjuvant combining cationic vesicles and trehalose dibehenate (TDB), a mincle receptor agonist, has been evaluated against MPL–plus–QS-21 formulations (17, 18), further highlighting the capacity of QS-21–based adjuvants to drive robust T_FH_ responses and humoral immunity. In this issue of the *JCI,* Parham Ramezani-Rad et al. (19) tested the saponin (QS-21)–MPLA nanoparticle (SMNP) adjuvant system, building on the principles of AS01 by incorporating an immune-stimulating complex–like (ISCOM-like) structure. This design enhanced innate immune activation and optimized antigen delivery to lymph nodes, key factors that bolster effective GC responses and the production of durable antibodies (20).

## A brief history of HIV vaccine efforts

Among the most prominent HIV vaccine studies, the RV144 trial employed a prime-boost strategy pairing a recombinant canarypox vector expressing HIV antigens with a gp120 Envelope (Env) protein boost formulated in Alum (21). While it showed modest efficacy in a low-risk population, protection waned, and follow-up studies with similar regimens using MF59 as the adjuvant failed to replicate these results (22). Despite its limitations, RV144 offered key insights, particularly the importance of both the magnitude and durability of antibodies targeting the HIV-1 Env for achieving protection against diverse viral strains. However, subsequent trials have consistently underscored a sobering challenge: current vaccine platforms fail to elicit long-lived, broadly neutralizing antibodies (nAbs) against difficult-to-neutralize (tier 2) HIV strains, which are the most representative of circulating viruses. Given the rarity of B cell precursors capable of targeting HIV epitopes, their successful maturation relies on pairing the right immunogen with an adjuvant to sufficiently activate innate immunity. This activation is critical for driving robust T_FH_ responses, which in turn support B cell proliferation, affinity maturation, and differentiation into plasma cells that produce durable, high-affinity antibodies, as well as memory B cells primed for rapid recall upon reexposure.

## Dose-dependent effect of SMNP adjuvant

Parham Ramezani-Rad et al. (19) investigated how varying doses of QS-21–containing SMNP (25, 50, 200, or 400 μg) influence T_FH_-driven B cell responses to BG505 MD39, a stabilized Env trimer optimized to enhance nAb epitope presentation (23). Groups of 6 adult rhesus macaques (balanced by sex) were primed at week 0 and boosted at weeks 10 and 24 with 100 μg BG505 MD39 Env protein given subcutaneously to ensure slow antigen release and nAb induction (24). Levels of inflammatory cytokines, including IL-6, monocyte chemotactic protein (MCP-1), and interferon protein 10 (IP-10), peaked on day 1, with the 400 μg dose inducing the strongest responses before returning to baseline by day 3. The occurrence of fevers mirrored this trend, resolving without adverse events.

Higher SMNP doses enhanced Env-specific CD4^+^ T cell responses in blood 2 weeks after priming. The 400 μg dose drove the strongest Th1 cell (CD40L^+^IFN-γ^+^) and T_FH_ (CD40L^+^OX40^+^CXCR5^+^PD-1^+^) differentiation, with memory Th1 cells persisting in all animals at higher frequencies compared with the 200 μg group. Although T_FH_ cell frequencies declined over time across all doses, they remained highest in the 400 μg group, whereas more pronounced attrition was observed in lower-dose groups. These findings underscore the role of higher adjuvant doses in sustaining robust CD4^+^ T cell memory.

Env-specific B cells in blood expanded rapidly after each boost, with the 400 μg group showing faster kinetics. Env-specific IgG levels in sera peaked 2 weeks after boosting and remained elevated in the 400 μg group for 14 weeks. Following the second boost, IgG levels in the 200 μg group rose to match those in the 400 μg group. In contrast to IgG, autologous nAbs against tier 2 viruses were detectable only in the 400 μg group after the first boost, while the 25 μg dose failed to elicit any nAbs. By 6 weeks after the final boost, nAb titers declined across all groups but remained higher in the 400 μg group. Bone marrow plasma cells (BMPCs), a hallmark of long-lived immunity, were assessed 13 weeks after the final boost. Both the 200 μg and 400 μg groups achieved 100% BMPC responses, with the 400 μg group exhibiting numerically higher frequencies. In contrast, some animals in the lower-dose groups failed to generate detectable BMPCs.

The ability of SMNP to generate BMPCs and memory B cells is encouraging, yet the study by Parham Ramezani-Rad et al. (19) highlights the persistent challenge of eliciting robust nAb responses. Protective efficacy is generally associated with nAb titers exceeding 1:500 (25). Only a subset of animals retained tier 2 nAbs at the memory time point, possessing titers below 1:500 even at the highest SMNP dose, which underscores the difficulty of overcoming this bottleneck in HIV vaccine development. Therefore, the protective value of the tier 2 nAb levels remains unclear. Strategies such as additional boosting may help increase nAb titers and durability. Moreover, priming with mRNA or viral vectors could offer a synergistic benefit, boosting antibody responses and persistence (26) while also inducing CD8^+^ T cell responses, which are often not stably generated with protein immunogens alone. In conclusion, QS-21 SMNP demonstrated strong, dose-dependent activation of T and B cell responses. The observed transient reactogenicity without adverse events underscores that short-term pain is a necessary trade-off for long-term gain in achieving durable immunity.

## Clinical implications

With phase I trials (e.g., HVTN 144) underway, this macaque study offers important insights into the impact of adjuvant dosing on immune responses, and is expected to inform future vaccine platform design (19). For instance, the HVTN 137A trial showed that 30% of participants who received BG505 SOSIP.664 gp140 formulated with the TLR7/TLR8 agonist 3M-052 in Alum developed autologous tier 2 nAbs (27). Building on these findings, it will be essential to assess whether N332-GT5, derived from BG505 MD39 and paired with SMNP in HVTN 144, can not only enhance these responses but also broaden nAb induction to include heterologous nAbs. Since GC responses are challenging to study in human studies, integrating data from animal models and human studies will be vital for fine-tuning prime-boost regimens and advancing efforts toward durable immunity.

Although a licensed HIV vaccine remains elusive, decades of research have driven breakthroughs such as mRNA technologies, which enabled the rapid development of COVID-19 vaccines. These advances may finally help overcome the unique challenges posed by HIV and other elusive pathogens.

## Version 1 04/15/2025

Electronic publication
